# The distinct development of stimulus and response serial dependence

**DOI:** 10.3758/s13423-024-02474-8

**Published:** 2024-02-20

**Authors:** Liqin Zhou, Yujie Liu, Yuhan Jiang, Wenbo Wang, Pengfei Xu, Ke Zhou

**Affiliations:** 1 Beijing Key Laboratory of Applied Experimental Psychology, National Demonstration Center for Experimental Psychology Education (Beijing Normal University), Faculty of Psychology, Beijing Normal University, Beijing, China; 2 Sino-Danish College, University of Chinese Academy of Sciences, Beijing, China; 3 State Key Laboratory of Brain and Cognitive Sciences, Institute of Biophysics, Chinese Academy of Sciences, Beijing, China

**Keywords:** Serial dependence, Development, Attractive bias, Repulsive bias, Perception

## Abstract

Serial dependence (SD) is a phenomenon wherein current perceptions are biased by the previous stimulus and response. This helps to attenuate perceptual noise and variability in sensory input and facilitates stable ongoing perceptions of the environment. However, little is known about the developmental trajectory of SD. This study investigates how the stimulus and response biases of the SD effect develop across three age groups. Conventional analyses, in which previous stimulus and response biases were assessed separately, revealed significant changes in the biases over time. Previous stimulus bias shifted from repulsion to attraction, while previous response bias evolved from attraction to greater attraction. However, there was a strong correlation between stimulus and response orientations. Therefore, a generalized linear mixed-effects (GLME) analysis that simultaneously considered both previous stimulus and response, outperformed separate analyses. This revealed that previous stimulus and response resulted in two distinct biases with different developmental trajectories. The repulsion bias of previous stimulus remained relatively stable across all age groups, whereas the attraction bias of previous response was significantly stronger in adults than in children and adolescents. These findings demonstrate that the repulsion bias towards preceding stimuli is established early in the developing brain (at least by around 10 years old), while the attraction bias towards responses is not fully developed until adulthood. Our findings provide new insights into the development of the SD phenomenon and how humans integrate two opposing mechanisms into their perceptual responses to external input during development.

**Supplementary Information:**

The online version contains supplementary material available at 10.3758/s13423-024-02474-8.

Recent experience can be a reliable indicator of the current state of the physical environment due to a general tendency for stability over short periods. Sensory input can be noisy and chaotic, but individuals are able to efficiently form more precise perceptions in a Bayesian manner through the integration of recent information with current sensory input (Kersten et al., 2004; Körding & Wolpert, 2004). In perception research, the sequential integration of current perceptions with those from the recent past is referred to as serial dependence (SD) or serial bias (Barbosa et al., 2020; Cicchini et al., 2018; Corbett et al., 2011;  Fischer & Whitney, 2014; Fritsche et al., 2020; Kim et al., 2020; Kiyonaga et al., 2017; Manassi & Whitney, 2022). The SD effect has been observed across a variety of perceptual qualities, including low-level features such as orientation (Cicchini et al., 2021; Fischer & Whitney, 2014); motion direction ( Fischer et al., 2020); numerosity (Cicchini et al., 2014; Fornaciai & Park, 2018); and high-level features, such as identification of faces (Liberman et al., 2014), emotions (Manassi et al., 2018), age, and gender (Manassi & Whitney, 2022).

The attractive SD, in which current perceptions are biased towards recent stimuli, is well documented, but the underlying cognitive mechanisms of the effect are a topic of ongoing debate. Some studies have reported that the previous stimulus can produce attractive SD, even in the absence of the previous response (J. Fischer & Whitney, 2014; Fornaciai & Park, 2018; Liberman et al., 2014; Manassi et al., 2018). However, others showed that the prior ignored (Rafiei et al., 2021) or nonreported stimulus produces a repulsive bias (Pascucci et al., 2019). Previous research has employed computational modelling or neuroimaging approaches to demonstrate the coexistence of a bias that repels the perceiver away from the previous stimulus and a bias that attracts the perceiver towards the previous response (Hajonides et al., 2023; Moon & Kwon, 2022; Pascucci et al., 2019; Sadil et al., 2023; Sheehan & Serences, 2022; Zhang & Luo, 2023). Researchers interpreted the repulsive SD as a form of visual adaptation (Pascucci et al., 2019) and the attractive SD as related to Bayesian inference (Fritsche et al., 2020; Sadil et al., 2023) and attentional priming (Kristjánsson, 2010). Despite these theoretical debates, both perspectives encourage considering the SD effects of the preceding stimulus and response independently.

Although previous research has explored the cognitive mechanisms of SD biases of the previous stimulus and/or response in adults, and psychiatric studies have observed abnormal biases towards the recent past in individuals with autism (Feigin et al., 2021; Lieder et al., 2019; Turbett et al., 2022), anti-N-methyl-D-aspartate receptor encephalitis (Stein et al., 2020), and schizophrenia (Stein et al., 2020), the developmental course of the effect remains unknown and possible variations in SD across age groups have yet to be explored.

To address this gap in the literature, the present study examined the SD effects of the previous stimulus and response across different developmental stages, from childhood (around 10 years) to early adulthood. The study employed an orientation reproduction task. The classical orientation reproduction paradigm utilizes multiple levels of relative orientation between Gabor patches in consecutive trials, with SD being described as a Derivative-of-Gaussian (DoG) like curve (J. Fischer & Whitney, 2014; Fritsche et al., 2020). However, this experiment is challenging for children, as it typically takes up to an hour to complete, requiring a significant amount of concentration. To address this issue, we simplified the experiment by reducing the levels of relative orientation between Gabor patches in two consecutive trials to three levels (−15°, 0°, and 15°), thereby reducing the duration of the experiment to 25 minutes. In addition, the −15°–15° range allowed for the use of linear models that would accurately fit the data and measure the regression coefficients of the preceding stimulus and response. A positive and negative regression coefficient indicated an attractive and repulsive bias, respectively. By comparing regression coefficients between age groups, we were able to examine the development of SD with age and assess the developmental trajectories of the SD produced by the previous stimulus and response.

## Methods

### Participants

A total of 127 participants took part in the study. Two child participants were excluded from the final analysis due to an average response time exceeding 10 s in one case, and an average absolute response error exceeding 30° in the other. As a result, the final analysis included 125 participants, comprising 46 children (mean age = 10.61 years, age range: 9–12; 19 females), 38 adolescents (16.61 years, 15–17; 23 females), and 41 early adults (21.73 years, 18–29; 27 females). All participants had normal or corrected-to-normal vision and normal nonverbal Raven IQ scores (Raven et al., 2000) at or above the 50^th^ percentile.

### Ethical statement

The study was conducted in accordance with the tenets of the 2013 revision of the Declaration of Helsinki, and the protocol was approved by the Institutional Review Board of Beijing Normal University. Informed consent to participation and publication was obtained from participants and, in those under 18, their parents or guardians.

### Materials and procedures

**Fig. 1:**
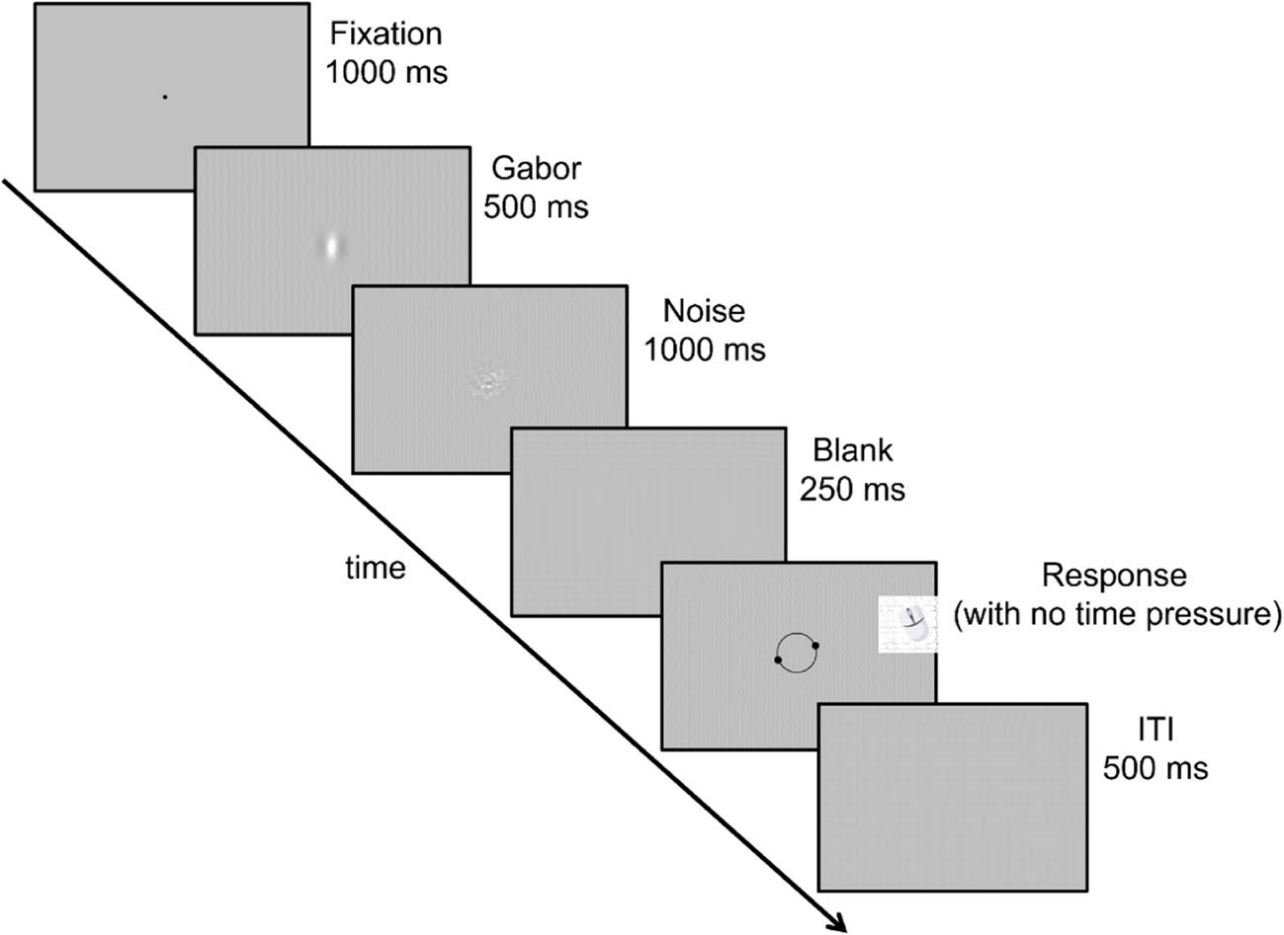
Example trial sequence on the orientation reproduction task. Participants viewed a Gabor patch presented in the center of a screen and subsequently reproduced the perceived orientation by adjusting the orientation of an imaginary line connecting two dots framed by a circle. ITI = intertrial interval

Participants viewed Gabor patches on a screen at a viewing distance of 60 cm. All stimuli were presented at the center of the screen. Each trial began with the presentation of a black fixation dot (diameter 0.5°) for 1,000 ms, followed by a Gabor patch (at a contrast of 25%) with a spatial frequency of 0.5 cycles per degree and a Gaussian envelope of 0.71° standard deviation (*s.d.*; Fig. 1). Each patch was presented for 500 ms. Following the presentation of each Gabor patch, a mask of white Gaussian noise, smoothed with a 3.3° *s.d.* Gaussian kernel and windowed by a 0.71° *s.d.* Gaussian envelope was presented for 1,000 ms to minimize negative aftereffects. After the mask and a further delay of 250 ms, a randomly oriented response stimulus appeared at the center of the screen, consisting of a black circular frame (diameter 2.8°) surrounding the location of the previous Gabor with two symmetrical dots (diameter 0.5°) marking the ends of an imaginary orientated line. Participants were asked to move a computer mouse to adjust the orientation of the response stimulus to that of the preceding Gabor stimulus and confirm the selected orientation by pressing the return key on the keyboard; this caused the response stimulus to disappear. After an intertrial interval (ITI) of 500 ms, the next trial began.

The difference in orientation between the Gabor stimuli in each trial and that in the previous trial was −15°, 0°, or 15°, with the three conditions counterbalanced within each block. The child and adult groups completed 40 trials per block, while the adolescent group completed 55 trials per block. Each participant completed three blocks in total. Prior to the experiment, participants were required to perform practice trials until their mean absolute error was below 9°.

### Data processing

Trials with a response error (i.e., reported orientation minus stimulus orientation) greater than 30° and the trials following them were removed from further analysis (3.3% of trials). The first trial of each block was also excluded from the analysis due to the lack of a preceding trial.

#### ***Conventional analysis***

To replicate previous SD findings, we first measured SD using a conventional approach. Using this approach, the contributions of the previous stimulus and response were separately evaluated. Statistical testing was performed using MATLAB R2022a (The MathWorks, Inc., Natick, MA, USA). Specifically, we first used the fitlm function of the MATLAB Statistics and Machine Learning Toolbox to make the response error for each trial a function of the orientation difference between the previous and current stimulus (previous Gabor orientation minus current Gabor orientation, namely, relative orientation of previous stimulus) in a within-subject analysis. The estimated regression coefficient, which indicated the effect of the previous stimulus on the current response, was then analyzed. A positive regression coefficient indicates that the current response is biased towards the previous stimulus orientation (an attractive effect), while a negative regression coefficient indicates a bias away from the previous stimulus orientation (a repulsive effect). Also, the greater the absolute value of the regression coefficient, the greater the attractive/repulsive SD. One-sample *t* tests were then performed on the regression coefficients for each age group to examine the significance of the influence of the previous stimulus in each group, and independent samples t-tests were conducted to assess the significance of the regression coefficient differences between groups. These statistical tests were two-tailed and Bonferroni-corrected for multiple comparisons (p_corr_). We further tested the reliability of these finding using a nonparametric randomization test. We performed 1,000 shuffling iterations to randomize the relative orientations of the previous stimulus within each block for each participant, and reran the conventional analysis. The significance of the influence of the regression coefficient of the previous stimulus was verified if it fell beyond the 95% confidential interval (CI) obtained from the shuffling iterations. The same approach was used to evaluate the SD effect of the previous response orientation on the current response.

#### ***Joint bias map***

Since the response orientation always fluctuated around the orientation of the Gabor stimulus, a strong correlation was apparent. Therefore, fitting the response errors, such that they were functions of just the previous stimulus or response orientation, was deemed an insufficiently accurate reflection of the isolated effects of the previous stimulus or response. To gain a more comprehensive understanding of the development of these effects, a further examination of the effects of the previous stimulus or response relative to the current stimulus at each orientation level was conducted.

While there were three discrete relative orientations of the previous stimulus (15°, 0°, and 15°), the relative orientations of the previous response were continuously distributed from −70° to 70°. To facilitate the analysis, the relative orientations of the previous response were divided into 29 nonoverlapping bins of 5° width. Subsequently, for each participant, the average response error for each level/bin of relative orientation of the previous stimulus and response was calculated to construct a two-dimensional joint bias map (Moon & Kwon, 2022). The *x*- and *y*-axes of this joint bias map represented the relative orientation of the previous response and stimulus, respectively. Colors on the map represented response errors, with blue and yellow indicating negative and positive response errors, respectively. To visualize the isolated effects more clearly, the response errors were further plotted as functions of the relative orientation of the previous stimuli at each relative orientation bin of previous responses. They were also plotted as functions of the relative orientation bins of previous response at each level of relative orientation of previous stimulus, based on the joint bias map of the mean values.

#### ***Generalized linear mixed-effects models***

The conventional analysis failed to deliver a comprehensive understanding of the SD, as it only accounted for each bias individually (Pascucci et al., 2023). To gain a more complete comprehension of the response errors, three generalized linear mixed-effects (GLME) models were devised and analyzed to identify the model that best explains the phenomenon (Zhang & Luo, 2022). Model 1 and Model 2 postulated that the previous stimulus and response, respectively, influence the current response, while Model 3 proposed that the previous stimulus and response simultaneously influence the current response. The equations for these models are as follows:

1 $$\mathrm{ERR }\sim 1 + {\mathrm{\Delta S}}_{{\text{prev}}}+ (1 + {\mathrm{\Delta S}}_{{\text{prev}}}|{\text{participant}});$$

2 $${\mathrm{ERR }\sim 1 +\mathrm{ \Delta R}}_{{\text{prev}}}{+ (1 +\mathrm{\Delta R}}_{{\text{prev}}}|{\text{participant}});$$

3 $$\mathrm{ERR}\sim1+{\mathrm\Delta\mathrm S}_\text{prev}+{\mathrm\Delta\mathrm R}_\text{prev}+(1+{\mathrm\Delta\mathrm S}_\text{prev}+{\mathrm\Delta\mathrm R}_\text{prev}\vert\text{participant}),$$

where ERR denotes the response error for the current trial; ΔS_prev_ and ΔR_prev_ denote the fixed effects of the relative orientation of the previous stimulus and response, respectively; and (1 + ΔS_prev_ + ΔR_prev_|participant) represents the random effects of the participants. These models were fitted using MATLAB’s fitglme.m function for normal distributions and the identity link function. Model performance was evaluated using the Bayesian information criterion (BIC). The ΔBIC of the models was calculated by subtracting the BIC of Model 3. The model with the lowest ΔBIC value was deemed the winning model. In line with conventional analyses, we further enhanced the reliability of the results by performing 1,000 shuffling iterations to randomize the relative orientations of the previous stimulus and response within each block for each participant, respectively, and reran the GLME analysis. The significance of the results was verified if they fell beyond the 95% CIs obtained from the shuffling iterations.

To investigate the development of the SD effect of the previous stimulus and response simultaneously, the fixed effects parameters of the winning model were compared between age groups using two-tailed independent-sample *t* tests that were Bonferroni corrected for multiple comparisons.

## Results

### Conventional analysis of the development of serial dependence

**Fig. 2:**
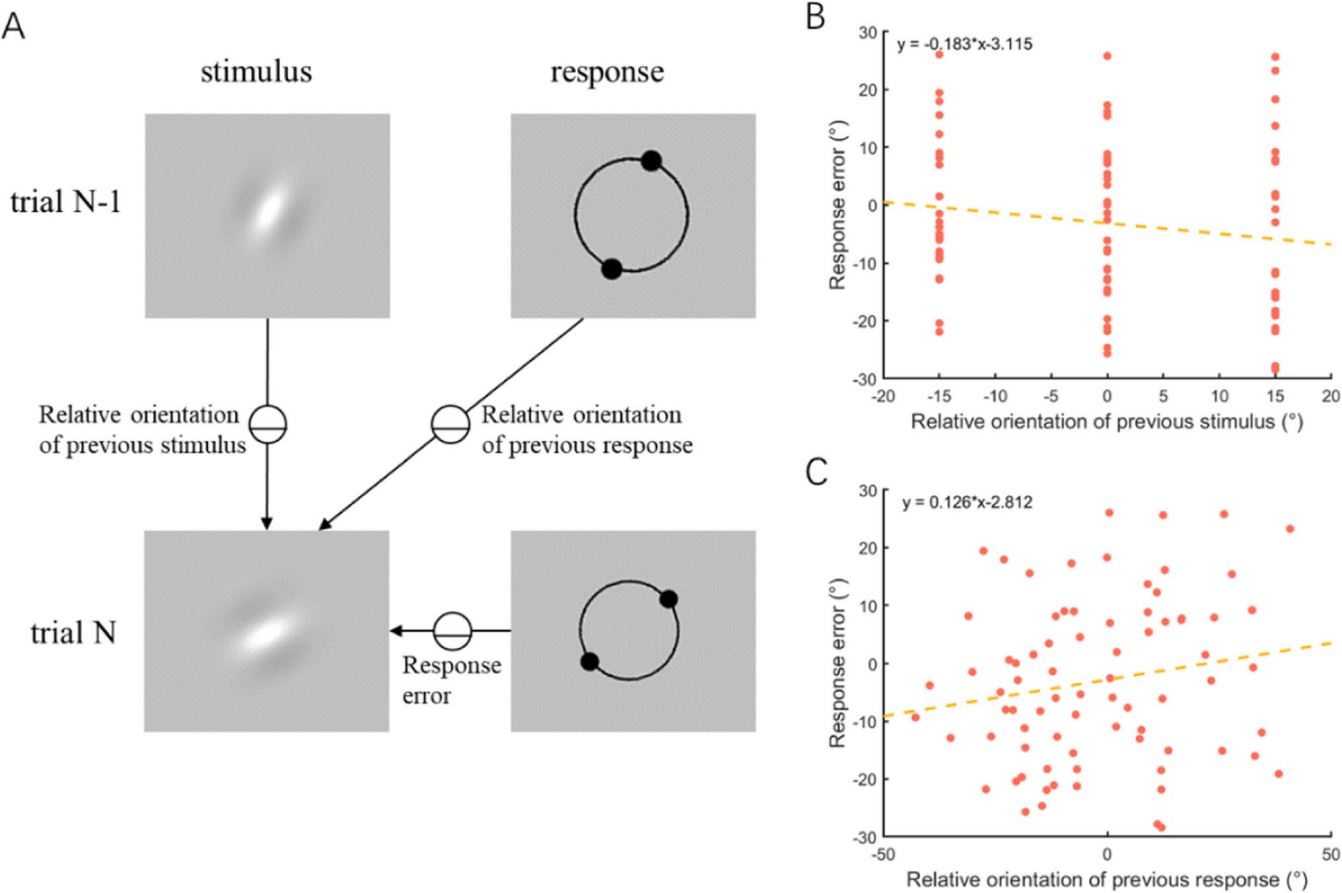
Serial dependence calculated using conventional analysis in one example participant.** A** Stimuli and responses in successive trials. Relative orientation of previous stimulus: previous Gabor orientation minus current Gabor orientation. Relative orientation of previous response: previous response orientation minus current Gabor orientation. Response error: current response orientation minus current Gabor orientation. **B** Illustration of the response error in relation to the relative orientation of the previous stimulus for one participant. The slope of the linear function is the participant’s regression coefficient of previous stimulus. **C** Illustration of the response error in relation to the relative orientation of the previous response for one participant. The slope of the linear function is the participant’s regression coefficient of the previous response. (Color figure online)

**Fig. 3:**
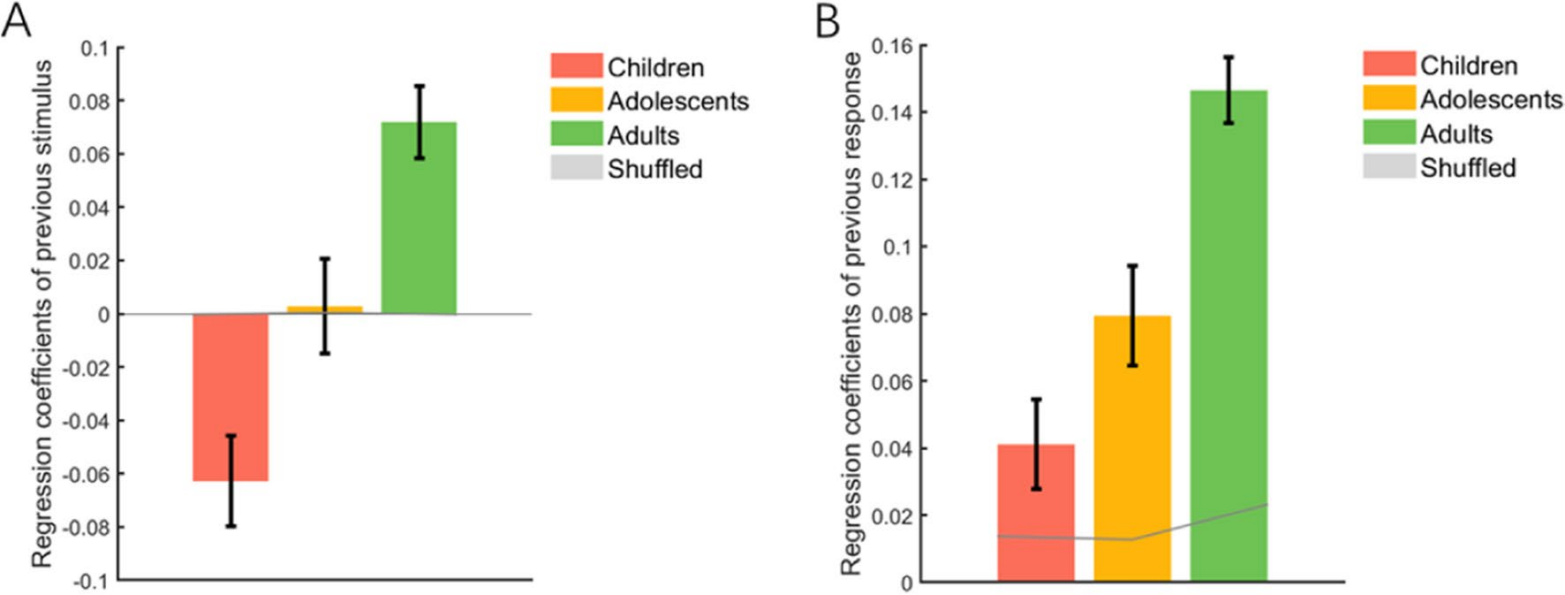
Results of conventional data analysis of the performance of three age groups on the orientation reproduction task. **A** The regression coefficients of the previous stimulus on current response errors. A positive regression coefficient indicates that the current response is biased towards the previous stimulus orientation (an attractive effect), while a negative regression coefficient indicates a bias away from the previous stimulus orientation (a repulsive effect). Also, the greater the absolute value of the regression coefficient, the greater the attractive/repulsive SD. **B** The regression coefficients of the previous response on current response errors. In each subgraph, the dark gray line and light gray shadow represent the mean values (e.g., mean regression coefficients of previous stimulus for Fig. 3A; mean regression coefficients of previous response for Fig. 3B) and their corresponding 95% CIs, respectively, calculated from 1,000 shuffling iterations. Error bars represent ±1 standard error. (Color figure online)

We first conducted a conventional analysis of our data to separately replicate the previously reported SD effects of previous stimulus and response. The response errors were individually modeled as linear functions of the relative orientation of the previous stimulus or response to the current stimulus. Figure 2 showed SD calculated using conventional analysis in an example participant. The group-level results are shown in Fig. 3. In line with previous findings (J. Fischer & Whitney, 2014; Moon & Kwon, 2022; Pascucci et al., 2019), adults were found to exhibit significant SD attraction biases towards both previous stimulus, *t*(40) = 5.32, p_corr_ < .001, Cohen’s *d* = 0.81, and previous response, *t*(40) = 14.84, p_corr_ < .001, Cohen’s *d* = 2.27. The results were additionally validated through a randomization test, as they fell beyond the 95% CIs obtained from 1,000 shuffling iterations.

However, a more detailed examination of the data revealed that the effects of the previous stimulus and response varied between age groups. One-sample *t* tests revealed that the previous stimulus produced repulsive effects in children, *t*(45) = −3.71, p_corr_ = .002, Cohen’s *d* = −0.54, but not in adolescents, *t*(37) = 0.16, p_uncorr_ = .88, Cohen’s *d* = 0.02 (Fig. 3A). These findings were further substantiated by randomization tests. Independent sample t-tests further revealed significant differences in the effect between each two of the three age groups—children vs. adolescents: *t*(82) = −2.66, p_corr_ = .03, Cohen’s *d* = −0.58; children vs. adults: *t*(85) = −6.11, p_corr_ < .001, Cohen’s *d* = −1.30; and adolescents vs. adults: *t*(77) = −3.12, p_corr_ = .009, Cohen’s *d* = −0.70, with the effect shifting from repulsive to attractive with increasing age.

Like adults (Fig. 3B), both children, *t*(45) = 3.07, p_corr_ = .012, Cohen’s *d* = 0.45, and adolescents, *t*(37) = 5.37, p_corr_ < .001, Cohen’s *d* = 0.85, exhibited a strong attractive bias towards the previous response. Further independent-sample *t* tests revealed that the attractive bias was much stronger in adults than in children, *t*(85) = 6.23, p_corr_ < .001, Cohen’s *d* = 1.33, and adolescents, *t*(77) = 3.83, p_corr_ < .001, Cohen’s *d* = 0.85. However, there was no difference in the strength of the attractive bias between children and adolescents, *t*(82) = 1.93, p_corr_ = .174, Cohen’s *d* = 0.42. These results indicated that the attractive bias effect of previous responses increased with age.

The developmental trajectories of biases of both previous stimulus and response were replicated when age was treated as a continuous rather than a categorical variable, and correlated with the regression coefficients of previous stimulus and response (Fig. S1).

However, because the relative orientation of response was strongly correlated with that of stimulus (Pearson’s *r* = .81 ± 0.06, mean ± *s.d.*), the conventional approach is unable to accurately isolate the unique contributions of the previous stimulus and response to SD effects. Therefore, it is more appropriate to examine the effects of the previous stimulus and response while controlling for the relative orientation of the previous response or stimulus.

### The joint bias of the previous stimuli and responses

**Fig. 4:**
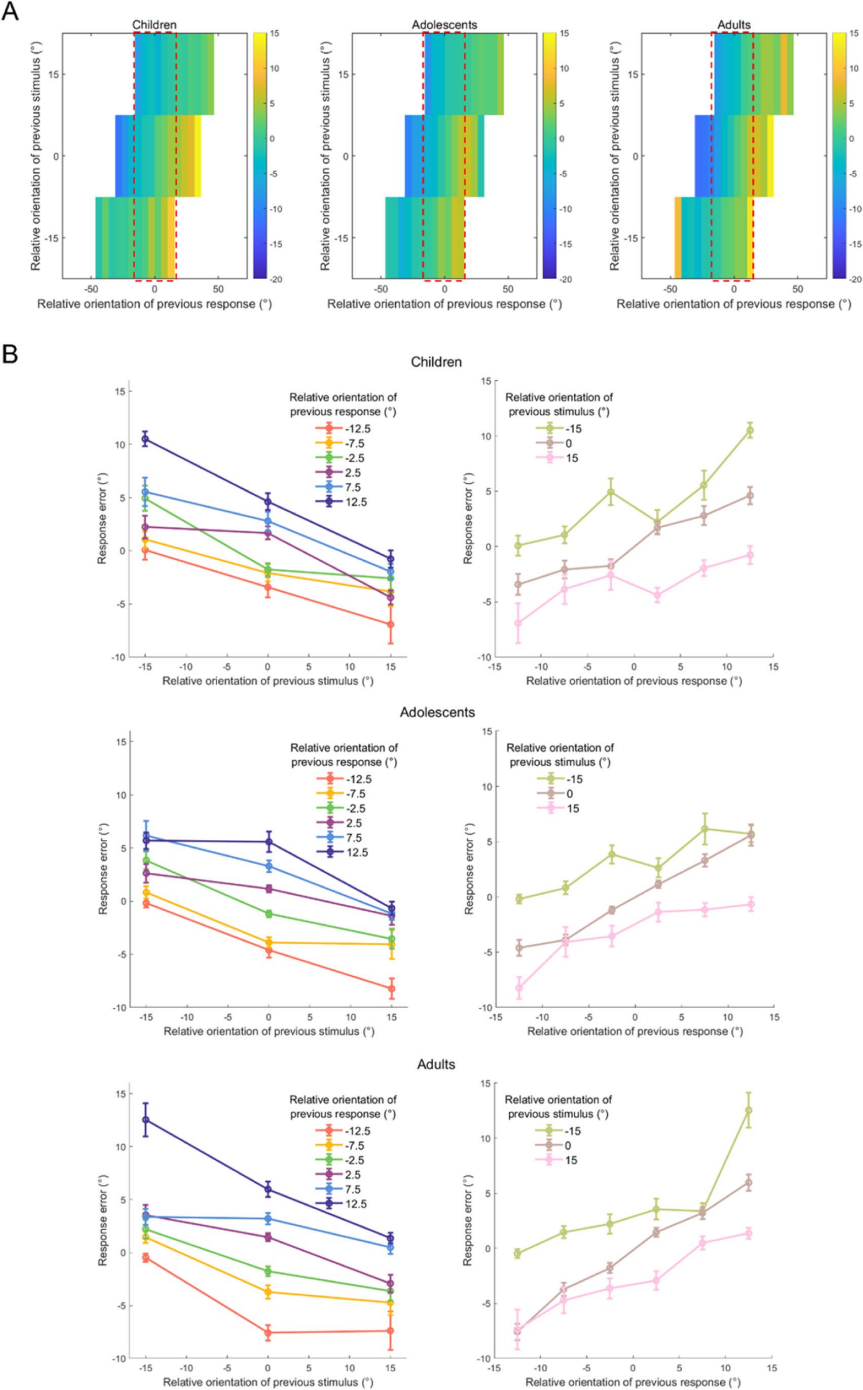
Results of joint bias analysis of the performance of three age groups on the orientation reproduction task. **A** Average joint bias map of the averages for each age group. Response errors are plotted as functions of the relative orientation of the previous stimulus and response (compared with the current stimulus). The colors represent the value of response errors. The positive values of relative orientations and response errors are represented as counterclockwise differences and the negative values as clockwise differences. More details are given in the Methods section. **B** Response errors are plotted as functions of the relative orientation of the previous stimulus conditioned by the relative orientation of the previous response (*left*) and as functions of the relative orientation of the previous response conditioned by the relative orientation of the previous stimulus (*right*). The data plotted are from the area inside the red dashed line box in the joint bias maps shown in (**A**). A positive slope indicates that the current response is biased towards the previous stimulus (or response) orientation (an attractive effect), while a negative slope indicates a bias away from the previous stimulus (or response) orientation (a repulsive effect). Also, the greater the absolute value of the slope, the greater the bias. Error bars represent ±1 standard error. (Color figure online)

To isolate the effects of the previous stimulus and response, response errors were calculated for each participant at various relative orientation levels of the previous stimulus and various relative orientation levels of the previous response. This resulted in a two-dimensional matrix of mean response errors across participants, as a function of previous stimuli and responses (Fig. 4A). In contrast to the findings of our conventional analysis, when the relative orientation of the previous response was fixed, the response error became more negative as the relative orientation of the previous stimulus increased. This was true of the joint bias maps for all three age groups. Conversely, when the relative orientation of the previous stimulus was fixed, the response error became more positive as the relative orientation of the previous response increased.

To provide a clearer visualization of this phenomenon, six relative orientation bins of previous responses with response error values at all levels of relative orientation to the previous stimulus were selected. Participants’ response errors were plotted as functions of these bins and the relative orientations of the previous stimulus. This confirmed the repulsive and attractive bias effects of the previous stimulus and response in all three groups, as shown in Fig. 4B. The discrepancy between the results of the joint bias map and the conventional analysis indicated that separately considering each factor was insufficient, and emphasized the importance of simultaneously taking both factors into account.

### Development of isolated serial dependence of the previous stimulus and response

**Fig. 5:**
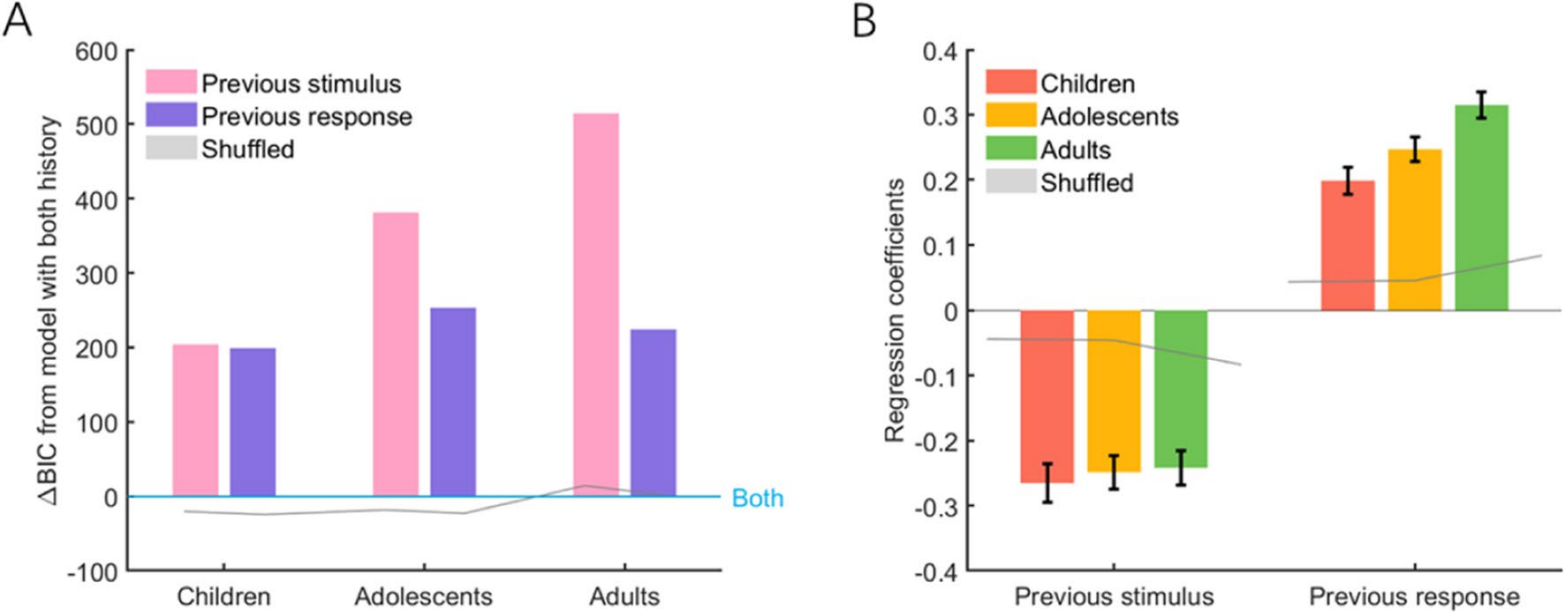
Results of generalized linear mixed-effects analysis of the performance of three age groups on the orientation reproduction task.** A** Model comparison results. The ΔBIC of Model 1 (previous stimulus) and Model 2 (previous response) compared with that of Model 3 (previous stimulus + previous response). Positive ΔBIC values indicate the model is worse than Model 3, and negative ΔBIC values indicate the model is a better fitting model than Model 3. **B** The regression coefficients for the previous stimulus and response. Negative regression coefficient values represent repulsive bias, and positive values represent attractive bias. Moreover, the larger the absolute value of the regression coefficient is, the larger the bias is. In each subgraph, the dark gray line and light gray shadow represent the mean values (e.g., mean ΔBICs for Fig. 5A; mean regression coefficients for Fig. 5B) and their corresponding 95% CIs, respectively, calculated from 1,000 shuffling iterations. Error bars represent ±1 standard error. ΔBIC = delta Bayesian information criterion. (Color figure online)

Three GLME models were constructed to account for the effects of the previous stimulus and response to the current response error. The first two models considered the effects of either the previous stimulus or response, while the third model considered the combined effects of both. For each model, the BIC was computed and then subtracted from the BIC value of Model 3 to compare the model’s performance with that of Model 3. As shown in Fig. 5A, both Models 1 and 2 exhibited poorer performance than Model 3 across all three age groups—Model 1: ΔBIC = 203.98 (children), 381.45 (adolescents), 514.56 (adults); Model 2: ΔBIC = 199.18 (children), 253.44 (adolescents), 224.26 (adults). This observation aligned with the notion that preceding stimuli and responses jointly influence current responses, which was the case in previous computational modelling investigations that simultaneously considered the serial bias of the previous stimulus and response (Moon & Kwon, 2022; Pascucci et al., 2019; Sadil et al., 2023; Zhang & Luo, 2023). The Model 3’s status as the winning model across all age groups was further confirmed by the observation that the ΔBICs exceeded the upper boundary of the 95% CIs obtained from 1,000 shuffling iterations.

Model 3 revealed negative regression coefficients for the previous stimulus (coefficients ≤ −0.24, *t*s ≤ −9.06, *p*s < .001) and positive regression coefficients for the previous response (coefficients ≥ 0.20, *t*s ≥ 9.61, *p*s < .001) across all three age groups (Fig. 5B). Consistent with the findings from the joint bias analysis, the results confirmed the repulsive and attractive bias effects of the previous stimulus and response, respectively.

Furthermore, in Model 3, no significant differences were found between the previous stimulus regression coefficients of the age groups—children vs. adolescents: *t*(11068) = −0.43, p_uncorr_ = 0.670; children vs. adults: *t*(9712) = −0.59, p_uncorr_ = 0.556; adolescents vs. adults: *t*(10916) = −0.18, p_uncorr_ = 0.857. However, the regression coefficients for the previous response were significantly greater in adults than those of the other two age groups—children vs. adolescents: *t*(11068) = −1.70, p_corr_ = 0.267; children vs. adults: *t*(9712) = −4.04, p_corr_ < 0.001; adolescents vs. adults: *t*(10916) = −2.43, p_corr_ = 0.045.

To assess the reliability of the development in SD by GLME analyses, we reanalyzed the data with age as a continuous variable rather than categorical. Specifically, we introduced the interaction between participants’ age and the relative orientation of the previous stimulus or/and response in the GLME models. Model 3, considering both previous stimulus and response, remained the winning model (Fig. S1A). More importantly, we observed a significant interaction between age and previous response, but no significant interaction with previous stimulus (Fig. S2B and Table S1).

These results consistently suggest distinct developmental trajectories for the SD effects of the previous stimulus and response, with the repulsive bias of the previous stimulus stabilizing at least around the age of 10 and the attractive bias of the previous response continuing to develop at least into early adulthood.

## Discussion

The present study examined the development of SD using an orientation reproduction task. We initially replicated previously reported repulsive and attractive SD of the previous stimulus and response, respectively, using conventional analysis (J. Fischer & Whitney, 2014; Liberman et al., 2014; Moon & Kwon, 2022; Pascucci et al., 2019; Sadil et al., 2023). Our conventional analysis also found that both effects changed significantly over time, with the bias of the previous stimulus shifting from repulsion to attraction, and the bias of the previous response evolving from attraction to stronger attraction. Given the high correlation between the stimulus and response orientations, we went on to describe the isolated effects of the previous stimulus and response using a joint bias map. We found that the previous stimulus and response created a repulsive and attractive bias across all age groups, respectively. This conflicted with the findings of our conventional analysis. Comparisons of the performance of GLME models provided further quantitative confirmation that the model in which the contributions from both the previous stimulus and response were considered, provided the most accurate characterization of response errors compared with the models that considered only stimulus or response. A comparison of the regression coefficients of the winning GLME model revealed that the children around 10, adolescents, and adults displayed a similar repulsive bias towards the previous stimulus but differed in their attractive bias towards the previous response. These findings suggest that the developmental trajectories of the SD effects of the previous stimulus and response are distinct from one another.

The joint bias map and regression coefficients of the winning GLME model provided insight into the contributions of both the previous stimulus and response and revealed a repulsive bias towards the previous stimulus and an attractive bias towards the previous response. These results were in line with those of earlier studies that have simultaneously accounted for both factors (Moon & Kwon, 2022; Pascucci et al., 2019; Sadil et al., 2023; Zhang & Luo, 2022) and diverged from those with results obtained through conventional analysis. The superior performance of the GLME model that included both the previous stimulus and response demonstrates the importance of considering both variables together to accurately capture SD effects. Failure to do so may lead to misleading or inaccurate results.

In the winning model, there was no significant difference between the repulsive biases produced by the previous stimulus in children around 10, adolescents, and adults. The repulsive bias has been interpreted as a visual adaptation effect (Pascucci et al., 2019; Sadil et al., 2023) that improves visual performance by reducing sensitivity to adapting stimuli but not to novel stimuli (Alink et al., 2018; Clifford et al., 2007; Kohn, 2007). Although adaptation has traditionally been thought to require several seconds of adaptation duration, recent research has shown that it can occur in as little as 10s of milliseconds (Glasser et al., 2011; Kanai & Verstraten, 2005; Webster, 2015). Thus, it can be inferred that the stimulus-induced repulsive SD observed in the current study, in which the stimulus was presented for several hundred milliseconds, is a result of perceptual adaptation. Furthermore, the development of the repulsive bias towards the previous stimulus appears to stabilize at least around the age of 10. This is consistent with previous research that has shown adaptation aftereffects to have almost fully developed by this age. For example, studies have found that 8-year-old children already exhibit comparable adaptation effects for facial identification to adults (Nishimura et al., 2008; Pimperton et al., 2009) and vibrotactile adaptation stabilizes at 10 years of age (Domenici et al., 2022). These findings suggest that the two adaptation effects have similar developmental trajectories. In addition, to better understand when the stimulus-induced SD effect reaches maturity, it will be crucial for future studies to examine how this effect develops in children younger than 10 years old.

Unlike the repulsive bias towards the previous stimulus, the attractive bias towards the previous response was not fully developed in our sample until adulthood. The attractive bias is consistent with the Bayesian inference proposal that perceptual decisions are integrations of prior belief and current sensory evidence (Kersten et al., 2004; Körding & Wolpert, 2004; Summerfield & Parpart, 2022; Weiss et al., 2002). This notion has been evaluated in various studies using Bayesian observer models (Cicchini et al., 2014, 2018; Fritsche et al., 2020; van Bergen & Jehee, 2019). Research has shown children as young as four to be capable of Bayesian inference when making behavioral choices (Gopnik et al., 2004; Gopnik & Wellman, 2012; Kushnir & Gopnik, 2007). Other developmental research has demonstrated that prior context has a weaker influence on the decisions of children than those of adults in relation to visual search efficiency (Yang & Merrill, 2015) and (absolute) width (Sciutti et al., 2014), and the Bayesian inferences of children are less efficient than those of adults (Chambers et al., 2018; Schulze & Hertwig, 2022). Additionally, adolescents have a higher tolerance for uncertainty (Blankenstein et al., 2016; Tymula et al., 2012) and have been found to exhibit elevated learning and exploration rates than adults in noisy but stable environments, suggesting an overestimation of environmental volatility and weaker effects of priors (Jepma et al., 2020). Therefore, it is likely that the ability to conduct Bayesian inference develops with age, which aligns with the development of the attractive bias towards the previous response.

There were also certain limitations to this study. First, while stimuli are objective physical quantities, responses involve complex aspects, at least including perceptual distortions and response execution. Determining the specific stages responsible for the attractive bias of the previous response remains a critical challenge. This underscores the necessity for developing an experimental design methodology that can effectively disentangle perception from response choice in future research. Second, while our experimental design of three relative orientations effectively met the need for a child-friendly and time-efficient approach, addressing the challenges inherent in the classical orientation reproduction paradigm in terms of duration and children’s concentration levels, this simplification may have inadvertently overlooked factors such as peripheral biases, feature tuning, and the potential for edge effects. Future research in this area should take these confounding variables into account as much as possible.

To the best of our knowledge, this is the first study to investigate the development of SD. Through the utilization of three age groups, we effectively distinguished the SD’s repulsive and attractive biases produced by previous information (stimulus, response). We revealed distinct developmental trajectories for these two biases, with the repulsive effect of the previous stimulus being fully developed at least by around the age of 10 but the attractive effect of the previous response not being fully developed until adulthood. These findings provide new developmental insights into the nature of the SD and help to understand how human incorporates two opposing mechanisms to adapt in response to new experiences in the environment during development. Future research is necessary to explore the specific neural processes involved and their relationships to the distinct developmental trajectories observed in this study.

## Supplementary information

Below is the link to the electronic supplementary material.

Supplementary file1 (DOCX 319 KB)

## Data Availability

All materials and data have been made publicly available via the Open Science Framework (https://osf.io/3gn9j/).
