# Supplementary material for: The distinct development of stimulus and response serial dependence
Source: Psychon Bull Rev. 2024 Feb 20;31(5):2137–47. doi: 10.3758/s13423-024-02474-8 (PMC11543724; doi:10.3758/s13423-024-02474-8)
Supplement: Supplementary file 1 — Supplementary file1 (DOCX 319 KB) [file 13423_2024_2474_MOESM1_ESM.docx]

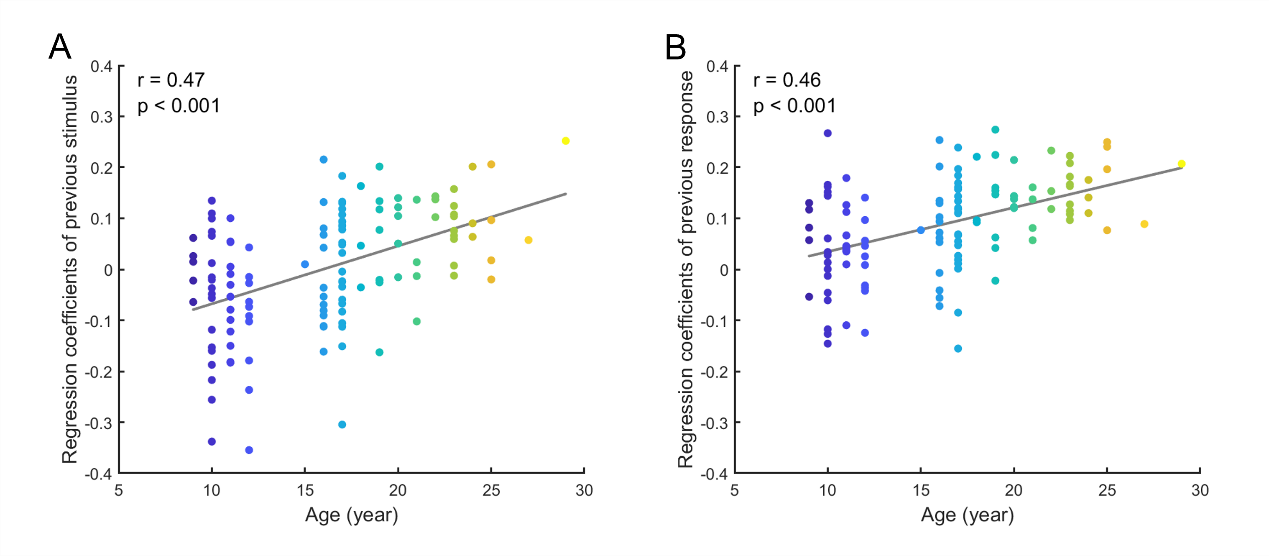


**Fig. S1. The correlation between age and regression coefficients of both the previous stimulus (A) and response (B)** **in conventional analysis.**


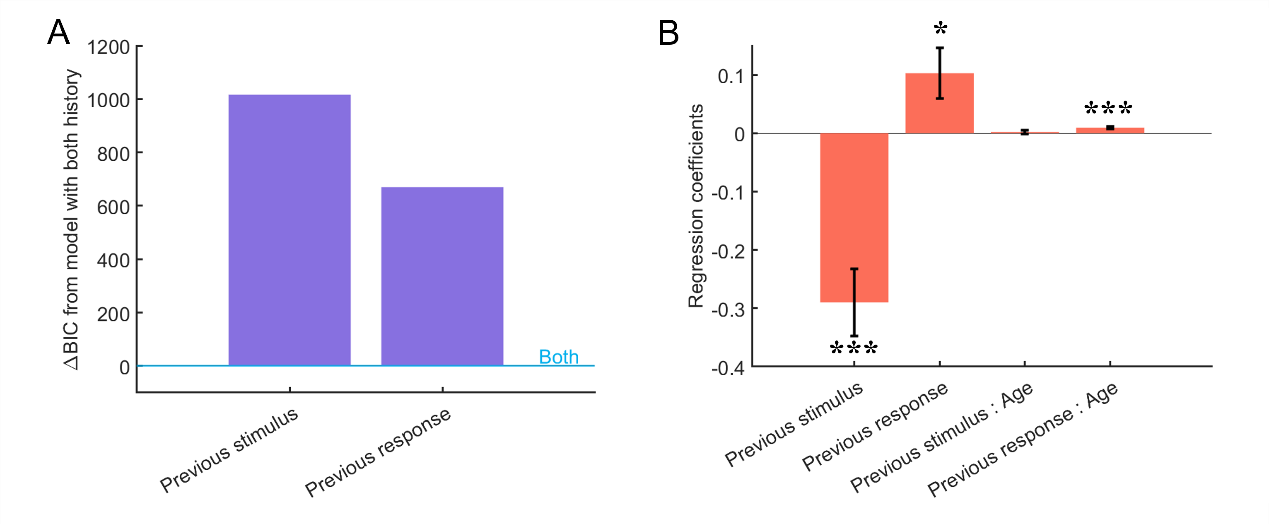


**Fig. S2. Results of GLME analysis including the interaction between participants’ age and the relative orientation of the previous stimulus and response in the GLME model.** (A) Model comparison results. The ΔBIC of model 1 (previous stimulus) and model 2 (previous response) compared to that of model 3 (previous stimulus + previous response). Positive ΔBIC values indicate the model is worse than model 3, and negative ΔBIC values indicate the model is a better fitting model than model 3. (B) The regression coefficients for the previous stimulus and response, and their interaction with age. Error bars represent ±1 standard error. *: 0.01<p≤0.05; ***: p<0.001.

Here are the equations for the three models, incorporating the interaction the interaction between participants’ age and the relative orientation of the previous stimulus or/and response in the GLME model:

(1) ERR ~ 1 + ΔS_prev_ + ΔS_prev_:Age + (1 + ΔS_prev_ + ΔS_prev_:Age |participant);

(2) ERR ~ 1 + ΔR_prev_ + ΔR_prev_:Age + (1 + ΔR_prev_ + ΔR_prev_:Age|participant);

(3) ERR ~ 1 + ΔS_prev_ + ΔR_prev_ + ΔS_prev_:Age + ΔR_prev_:Age + (1 + ΔS_prev_ + ΔR_prev_ + ΔS_prev_:Age + ΔR_prev_:Age|participant),

where ERR denotes the response error for the current trial; ΔS_prev_ and ΔR_prev_ denote the fixed effects of the relative orientation of the previous stimulus and response, respectively; ΔS_prev_:Age and ΔR_prev_:Age denote the fixed effects resulting from the interaction of age with the relative orientation of the previous stimulus and response, respectively.

**Table S1. Estimation results of coefficients in model 3** **including the interaction between participants’ age and the relative orientation of the previous stimulus and response in the GLME model.**

| Regressors | Estimate | SE | t | df | p | CI_lower_ | CI_upper_ |
| --- | --- | --- | --- | --- | --- | --- | --- |
| (Intercept) | 0.062 | 0.130 | 0.47 | 15846 | 0.636 | -0.194 | 0.317 |
| Previous stimulus | -0.290 | 0.058 | -5.03 | 15846 | <0.001 | -0.403 | -0.177 |
| Previous response | 0.103 | 0.043 | 2.40 | 15846 | 0.017 | 0.019 | 0.187 |
| Previous stimulus : Age | 0.002 | 0.003 | 0.65 | 15846 | 0.518 | -0.004 | 0.009 |
| Previous response : Age | 0.009 | 0.003 | 3.72 | 15846 | <0.001 | 0.004 | 0.014 |

SE: Standard error. CI: 95% confidential interval.
